# Diet, lifestyle factors, comorbidities, and hepatocellular carcinoma risk in a middle eastern country: a case-control study

**DOI:** 10.1186/s12885-024-12409-0

**Published:** 2024-06-06

**Authors:** Shaimaa Almohaid, Saeed Akhtar

**Affiliations:** Department of Community Medicine and Behavioural Sciences, College of Medicine, Kuwait University, PO Box 24923, Safat, 13110 Kuwait

**Keywords:** Hepatocellular carcinoma, Risk factors, Nonalcoholic fatty liver disease, Non-steroid anti-inflammatory drugs, Olive oil, Milk, And milk substitutes

## Abstract

**Background:**

Hepatocellular Carcinoma (HCC) can be classified as one of the most common malignancies worldwide. There is scarcity of the published data on the risk factors for HCC in the Gulf Cooperation Council countries specifically Kuwait. Therefore, this case-control study sought to examine the risk factors associated with HCC in Kuwait.

**Methods:**

Fifty-three histopathologically confirmed HCC cases were recruited from the Kuwait Cancer Control Center Registry. One hundred ninety-six controls (1:4 ratio) were selected from medical and/ or surgical outpatient’s clinics at all six public hospitals of Kuwait. A structured questionnaire was used to collect the data both from cases and controls through face-to-face interviews. A multivariable logistic regression model was fitted to the case-control data. Adjusted odds ratios (OR_adj_) and their 95% confidence intervals (CI) were computed using the parameters’ estimates of the final model and used for interpretation of the model.

**Results:**

The HCC cases compared with the controls were 41.6 times more likely to have had the history of non-alcoholic fatty liver disease (NAFLD) (OR_adj_ = 41.6; 95% CI: 8.9–193.5; *p* < 0.001). The cases compared with the controls were more likely to have reported the history of heavy alcohol drinking (OR_adj_ = 14.2; 95% CI: 1.2–173.4; *p* = 0.038). Furthermore, compared with the controls, the HCC cases tended to frequently consume milk and/or milk substitutes (≥ 3 glass/ week) (OR_adj_ = 7.2; 95% CI: 1.2–43.4). Conversely however, there was a significant protective effect if the participants reportedly have had regularly used olive oil in their routine diet as a source of fat (OR_adj_ = 0.17; 95% CI: 0.04–0.80) or regularly used non-steroid anti-inflammatory drugs (NSAIDs) (OR_adj_ = 0.20; 95% CI: 0.05–0.71).

**Conclusions:**

This study showed that heavy alcohol consumption, NAFLD history, and excessive consumption of milk/ milk substitutes were associated with a significantly increased HCC risk. Conversely however, regular use of olive oil in the diet as a source of fat or regular use of NSAIDs had a significantly protective effect against HCC risk. Adapting healthy dietary habits and preventing/ treating NAFLD may minimize the HCC risk. Future research with a larger sample size may contemplate validating the results of this study and unraveling additional risk factors contributing to HCC risk. The resultant data may help design and implement evidence-based educational programs for the prevention of HCC in this and other similar settings.

## Introduction

Hepatocellular carcinoma (HCC) is one of the major types of primary liver cancer with an estimated 75% of all liver cancers [1]. Annually about seven million people die of HCC worldwide, making it the third leading cause of cancer-related deaths [2, 3]. HCC is the 5th most common cancer among men and the 9th most common cancer among women [3]. Recent global statistics revealed that the HCC incidence rate (per 10^5^person-years) and HCC related-mortality rates (per 10^5^person-years) were substantially higher among men (14.1, 12.9) than women (5.2, 4.8) [3]. Furthermore, HCC incidence rate varies substantially across the globe [4]. Asia and Africa have the highest incidence rate (per 10^5^ person-years) of HCC ranging between 3 and 26 in the world [5]. In East Asia, Magnolias has the highest HCC incidence rate (93.7 per 10^5^ person-years). However, China has the greatest number of HCC cases because of both an increased incidence rate (18.3 per 10^5^ person-years) and with the world’s largest population (1.4 billion). Between 1987 and 2012, a decline in the HCC incidence was observed in many Asian countries [6]. Conversely however, an increase HCC incidence was reported from India, Americas, Oceania, and most of the European countries [7]. In the Middle Eastern countries, the HCC prevalence is lower compared to sub-Saharan Africa and some Far East countries [4]. However, the HCC prevalence in the Middle Eastern countries has also been increasing and mostly among men than women [8] Over the past decade, the HCC incidence in the Middle Eastern countries has increased dramatically with more pronounced increase in Saudi Arabia (4.5 per 10^5^ person-years) and Egypt (26.4 per 10^5^ person-years) [9]. In Kuwait, the incidence rate of primary liver cancer is 11.7 per 10^5^ person-years and HCC-related mortality rate is 1.8 per 100,000 person-years [4].

Most frequent risk factors for HCC worldwide include lifestyle factors (tobacco smoking, heavy alcohol drinking, consumption of food contaminated with aflatoxin (AFB1), obesity), comorbidities (infection with hepatitis B virus (HBV), hepatitis C virus (HCV), histological liver fibrosis and cirrhosis resulting from HBV and HCV infections, exposure to chemical hazard, diabetes mellitus (DM), genetic hemochromatosis, nonalcoholic fatty liver disease (NAFLD) [10–12]. Furthermore, a dose-response relationship has been reported between AFB1 and HCC [13]. Metabolic syndrome, insulin resistance, abdominal adiposity, atherogenic dyslipidemia and high blood pressure (hypertension) have also been implicated in increasing the HCC risk [14]. A meta-analysis has revealed that metabolic syndrome increases the HCC risk by approximately 81% [15]. Parenthetically, a very high prevalence (22.1%) of metabolic syndrome among adults in Kuwait has been reported [16]. In GCC (Gulf Cooperation Council) countries including Kuwait, relatively low prevalences of infections with of HBV (1.1–2.3%) [17, 18] and HCV (0.8 − 1.5%) [18, 19] have been recorded. Moreover, relatively recently it has been shown that *Helicobacter pylori*, microbiota, co-infection with HBV and hepatitis D virus, oral contraceptive pills and chronic kidney disease were also associated with an increased HCC risk [20, 21]. Over the past decade, it has been shown that the use of statins, metformin, aspirin, and coffee consumption have protective effects against HCC risk [22]. However, there is a paucity of the published data on the risk factors profile for HCC risk in the middle eastern countries including Kuwait. Therefore, the objective of this case-control study was to investigate the risk factors including diet, lifestyles factors and comorbidities associated with HCC risk in Kuwait.

## Methods

### Study design, setting, and participants

Organizationally Kuwait has six governorates with well-defined area of each governorate, which comprises several demarcated districts. Medical services in each governorate comprise a network of primary health-care clinics and a public hospital. Moreover, there are centralized specialty hospitals, including the Kuwait Cancer Control Centre (KCCC). KCCC is a specialized cancer treatment hospital equipped with the modern facilities for cancer diagnosis, treatment, and follow-up. KCCC is also a full member of the international Association of Cancer Registries (IACR). As per IACR guidelines, KCCC registry collects information concerning malignant neoplasm, as well as mortality data from the Vital and Health Statistics Division of Ministry of Health, Kuwait. Five main areas identified by the IACR are used while assessing the quality of KCCC registry data i.e., completeness of the coverage, completeness of detail of cancer cases, accuracy of said detail, accuracy of reporting, and the accuracy of interpretation. KCCC registry data are also used for research, policymaking, and health service planning, which indeed is dependent on the quality of KCCC database. The accuracy of KCCC registry data appears to be just as accurate as other cancer registries in some western countries including United States, United Kingdom, and Netherlands [23]. Almost all the suspected cancer patients are referred to KCCC. The cancer patients which are initially diagnosed and/or treated elsewhere are also referred to KCCC for further follow-up and treatment management. Health-care is provided free by the government to the nationals, whereas foreign residents must pay nominal fee to seek medical services [24, 25].

A hospital-based case-control study design was implemented to identify the potential risk factors associated with HCC risk. HCC cases registered with KCCC were enrolled. As noted above, suspected cancer patients are referred to KCCC for confirmatory diagnosis and treatment. The cancer patients diagnosed elsewhere are referred to KCCC for follow-up treatment. KCCC is equipped with the modern facilities for cancer diagnosis and treatment. The four controls per case (1:4 ratio) were selected from the general medical and/ or surgical out-patient clinics of the six main public hospitals from all the six governorates of Kuwait.

### Case definition, inclusion, and exclusion criteria

A patient of any age, sex (male or female) and nativity (Kuwait or non-Kuwaiti) with histological confirmed HCC diagnosis and treated at KCCC by medical oncologist and hepatologists was enrolled as a case. A patient diagnosed with HCC elsewhere and underwent follow-up for subsequent treatment at KCCC was also eligible for enrollment. A patient with previous diagnosis of cancer of any other body sites was excluded.

### Control definition, inclusion, and exclusion criteria

An individual of any age, sex (male or female), nativity (Kuwait or non-Kuwaiti) and without the prior history of HCC and/or cancer of any other body sites was enrolled as a control. Individual visiting an outpatient clinic in selected tertiary-care hospitals with minor complaint (minor trauma/injuries, upper respiratory tract infections, skin rash/ infection, headache, etc.) or accompanying such person was considered for enrollment as a control. The controls were enrolled from the selected tertiary-care hospitals as a sample of convenience by implementing the purposive sampling strategy. No frequency matching was carried out. Individual suffering from acute morbidity or short-term visitor to Kuwait was excluded.

### Exposure assessment

A structured questionnaire prepared based on the pertinent literature review [26–28], was used to collect the data both from cases and controls by a trained interviewer. Before actual use in the field, the questionnaire was pretested on 30 individuals demographically alike to our potential study participants. The questionnaire comprised questions which were grouped into four sections i.e., (a) socio-demographics including current age (years), age (years) at diagnosis, gender, marital status, nationality, educational attainment, occupation, governorate of residence. (b) potential exposures such as family history, history of current and/ or past morbidities including HBV and/ or HCV infection status (12 questions). Specifically, for the enrolled HCC cases, the history of infection with HBV and/ or HCV was based on their medical records. Whereas, for the controls’ status of infection with HBV and/ or HCV was based on self-reports in response to a question i.e., have you been ever diagnosed as positive for infection with HBV and/ or HCV based on the laboratory test recommended by a physician or your own suspicion of potential exposure to these viral infections. (c) history of weight (kg), height (cm), and physical activity level, past and current smoking (6 questions). (d) dietary habits and past diagnosis of fatty liver, fibrosis, cirrhosis, non-alcoholic fatty liver disease (NAFLD). Dietary habits include frequency of consumption of dietary items, and food frequency (17 questions). For the cases, histopathological information from medical files in KCCC Registry was sought. For the cases, the date of HCC diagnosis was considered as an index year and history of potential exposures was assessed prior to the end of that year. Similarly, for the four controls for a given case, a date of pseudo-diagnosis of HCC was constructed, which was the date of HCC diagnosis in the case. The controls were asked to report their exposures prior to the end of the index year (i.e., year of their pseudo-diagnosis of HCC). Data collection was carried out from August through November 15, 2022. The HCC cases diagnosed between January 1, 2018, and November 14, 2022, were included in the study.

### Statistical analysis

Descriptive statistics of sociodemographics were computed to characterize the cases and controls. Chi-squared analysis was used to examine the relationship between the potential risk factors and HCC. The unadjusted associations of sociodemographics and exposures with HCC status were quantified by using simple logistic regression analysis. The variables significantly (*p* ≤ 0.150) related with HCC status on the univariable analysis were considered for possible inclusion in the multivariable logistic regression analysis. A backward stepwise procedure was used to arrive at the final multivariate logistic regression model. The variables significantly (*p* < 0.05) related with the case-control status were retained in the final multivariable logistic regression model. To evaluate the goodness-of-fit of the final model, we used the Hosmer and Lemeshow test. Logistic regression assumes that there is little to no multicollinearity between independent variables (IVs), which essentially means IVs shouldn’t be too highly correlated with each other. Nonetheless, we examined multicollinearity by seeking and evaluating the correlation matrix of the coefficients of logistic model. The interaction between any of the two main effects in the final multivariable logistic model was not our interest in this study, therefore none was evaluated. The main effects included in the final multivariable logistic regression model were used to obtain the adjusted odds ratios (OR_adj_), their corresponding 95% confidence intervals (CI) and were used for the interpretation of the final model. The analysis was performed using the Statistical Package for Social Sciences (SPSS version 26).

For this case-control study, our computed sample size was 53 cases and 196 controls (case to controls ratio = 1:4). Furthermore, this sample size was large enough to achieve a study power of 80% to relate most of the potential exposures (having prevalence of 10% or higher in general population) and the HCC status with an OR of 2.5 or higher using logistic regression assuming 5% significance level (α). This sample size also accounted for potential refusals up to 10%.

### Ethics

Before the interview, a written informed consent was sought both from the cases and controls after explaining the study objectives. Confidentiality of the data was assured to the participants. The study protocol was approved (protocol number 1007/11/08/2022) by the Ethics Committee of Ministry of Health, Kuwait. Additionally, the study protocol was also approved (#: VDR/EC-4056/178) by the Ethics Committee of Health Sciences Center, College of Medicine, Kuwait University.

## Results

### Characteristics of the samples

The final study samples included 53 HCC cases and 196 controls. More HCC cases tended to be 60 years old or older than controls (79.2% vs. 45.9%). The response rate among the cases and the controls was 100% and 91.2% (196/215) respectively. The cited reasons for non-response among the controls were mainly ‘busy’ (5), I don’t like’ (8) and need to go somewhere (6). The distributions of sex and nativity were nearly the same both among cases and controls. A slightly higher proportion of the cases (100%) than controls (94.3%) were ever married. The higher proportions of cases than controls have had a low level of educational attainment (62.3% vs. 54.6%), unemployment (73.9% vs. 56.9%) and a total family income (Kuwaiti dinars per month) ≤ 1000 (62.2% vs. 48.5%) (Table 1).

**Table 1:** Characteristics of the study participants in a hospital-based case-control study of hepatocellular carcinoma (HCC) risk in Kuwait, 2022

Characteristics	HCC Cases (*n* = 53) *n* (%)	Controls (*n* = 196) *n* (%)	*p*-value
Age (completed years) at Study Enrollment			< 0.001
< 60 years ≥ 60 years	11(20.8) 42 (79.2)	106 (54.1) 90 (45.9)	
Gender			0.786
Male Female	40 (75.7) 13 (24.5)	144 (73.5) 52 (26.5)	
Nationality			0.697
Kuwaiti Non-Kuwaiti*	34 (64.2) 19 (35.8)	120 (61.2) 76 (38.8)	
Marital Status			0.081
Never Married Ever Married	- 51 (100.0)	11 (5.7) 182 (94.3)	
Education Level			0.318
Lower Education (≤ high school) Higher Education (University degree)	33 (62.3) 20 (37.7)	107 (54.6) 89 (45.4)	
Employment status			0.028
Employed Unemployed	14 (26.4) 39 (73.9)	84 (43.1) 111 (56.9)	
Total family income (KDs/ month)			0.124
≤ 1000 > 1000	33 (62.2) 20 (37.7)	95 (48.5) 101 (51.5)	
Governorate of residence			0.051
Al-Asimah Hawalli Jahra Ahmadi Mubarak Al-kabeer Farwaniya	10 (19.2) 20 (38.5) 4 (7.7) 4 (7.7) 6 (11.5) 8 (15.4)	13 (6.6) 68 (34.7) 12 (6.1) 27 (13.8) 44 (22.4) 32 (16.3)	

*Non-Kuwaiti include non-Kuwaiti Arab (18 (34%) cases, 72 (36.7%) controls and non-Kuwaiti non-Arab (1 (1.9%) case and 4 (2.0%) controls

Non-Kuwaiti Arab mostly comes from Egypt and non-Kuwaiti non-Arab from India, Bangladesh, Pakistan, Sri Lanka

### Chi-squared and univariable logistic regression analyses

Chi-squared analyses showed the distributions of various groups of variables and their association with the HCC status including sociodemographics (age, marital status employment status, total family monthly income), lifestyle factors (cigarette smoking, hookah smoking, physical activity, BMI), comorbidities (family history of HCC, history of diabetes mellites type 2, history of infection with HBV, history of infection with HCV, history of fibrosis, history of cirrhosis, history of NAFLD, history of regular use of NSAID, history of cardiovascular disease, history of statins use), and dietary patterns (frequency of fruits and vegetables consumption, frequency of use of milk and milk substitutes, alcohol drinking, regular use of olive oil, consumption of seafood, butter, ghee, cream, consumption of nuts, consumption of turmeric (Table 2). The unadjusted ORs (OR_unadjusted_) and their corresponding 95% CIs for the variables in relation to case-control status are in Table 3. Univariable logistic regression analysis showed, that of the demographics, age, total family income (Kuwaiti Dinars/month) were significantly related to case-control status (*p* < 0.05). From among the lifestyle factors and comorbidities, family history of HCC, history of infections with HBV, HCV, history of diagnosis of fibrosis, cirrhosis, NAFLD, cardiovascular disease, regular use of NSAIDs, frequency of milk or milk substitutes use, heavy alcohol drinking, regular use of olive oil as a source of fat, consumption of nuts, and consumption of turmeric were significantly (*p* < 0.05) related to the case-control status (Table 3).

**Table 2:** Chi-squared analysis of risk factors associated with hepatocellular carcinoma (HCC) status in a hospital-based case-control study, Kuwait 2022

	Cases (*n* = 53) No. (%)	Controls (*n* = 196) No. (%)		*p*-value
***Sociodemographic variables***				
Age (completed years) at enrollment in the study.				< 0.001
< 60 ≥ 60	11 (20.8) 42 (79.2)	106 (54.1) 90 (45.9)		
Sex				0.768
Female Male	13 (24.5) 40 (75.5)	52 (26.5) 144 (73.5)		
Marital status				0.127
Never married Ever married	0 (0.0) 51 (100.0)	11 (5.7) 182 (94.3)		
Nativity				0.697
Kuwaiti Non-Kuwaiti	34 (64.2) 19 (35.8)	120 (61.2) 76 (38.8)		
Employment status				0.028
Employed Unemployed	14(26.4) 39(73.6)	84 (43.1) 111 (56.9)		
Education				0.318
≤ high school ≥ university degree	33 (62.3) 20 (37.7)	107 (54.6) 89 (45.4)		
Total family income (Kuwaiti Dinars/month)				0.124
≤1000 > 1000	32 (60.4) 21(39.6)	95 (48.5) 101 (51.5)		
***Lifestyle factors and history of comorbidities***				
Body mass index (BMI) classification				0.514
Normal (< 18.5) Overweight (18.5 - < 25) Obese (≥ 25)	7 (28.0) 8 (32.0) 10 (40.0)	19 (21.3) 25 (28.1) 45 (50.6)		
Daily physical activity				0.197
Sedentary/ low activity Moderate activity Active	31 (58.5) 16 (30.2) 6 (11.3)	139 (71.3) 39 (20.0) 17 (8.7)		
Cigarette smoking				0.381
No Yes	38 (71.7) 15 (28.3)	128 (65.3) 68 (34.7)		
Hookah smoking				0.063
No Yes	50 (96.2) 2 (3.8)	168 (87.0) 25 (13.0)		
Family history of HCC				0.003
No	36 (70.6)	166 (87.8)		
Yes	15 (29.4)	23 (12.2)		
Parents’ blood relationship				0.637
No Yes	35 (66.0) 18 (34.0)	134 (69.4) 59 (30.6)		
History of diagnosis of HBV infection				< 0.001
No Yes	45 (86.5) 7 (13.5)	193 (99.0) 2 (1.0)		
History of diagnosis of HCV infection				< 0.001
No Yes	38 (71.7) 15 (28.3)	195 (99.5) 1 (0.5)		
History of diagnosis of fibrosis				< 0.001
No Yes	4 (7.5) 49 (92.5)	192 (98.0) 4 (2.0)		
History of diagnosis of cirrhosis				< 0.001
No Yes	2 (3.8) 51 (96.2)		192 (98.0) 4 (2.0)	
History of diabetes mellitus type II				0.094
No Yes	24 (45.3) 29 (54.7)		114 (58.2) 82 41.8)	
History of diagnosis of non-alcohol fatty liver disease				< 0.001
No Yes	16 (30.2) 37 (69.8)		163 (83.2) 33 (16.8)	
History of regular* use of nonsteroidal anti-inflammatory drugs				< 0.001
No Yes	45 (84.9) 8 (15.1)		112 (58.0) 81 (42.0)	
History of diagnosis of cardiovascular disease				0.003
No Yes	46 (86.8) 7 (13.2)		128 (65.6) 67 (34.4)	
Regular* use of statins				0.075
No Yes	34 (65.4) 18 (34.6)		101 (51.5) 95 (48.5)	
***Dietary variables***				
Frequency of red meat consumption				0.699
Never/ rare one or more days/week	13 (24.5) 40 (75.5)		53 (27.2) 142 (72.8)	
Frequency of fruits and vegetables consumption				0.011
Never/ rare 1-3 days/week > 3 days/week	2 (3.8) 6 (11.3) 45 (84.9)		37 (18.9) 31 (15.8) 128 (65.3)	
Frequency of use of milk and milk substitute				0.006
≤ 2 glass / week ≥ 3 glass/ week	8 (15.1) 45 (84.9)		68 (34.7) 128 (65.3)	
Frequency of eggs consumption (days/week)				0.524
Never/ rare 1–3 days/week > 3 days/week	17 (32.1) 18 (34.0) 18 (34.0)		48 (24.6) 78 (40.0) 69 (35.4)	
Heavy alcohol drinking				0.002
No Yes	42 (84.0) 8 (16.0)		188(96.9) 6 (3.1)	
Use of olive oil as a main source of fat				0.003
No Yes	40 (75.5) 13 (24.5)		102 (52.0) 94 (48.0)	
Consumption of processed and red meat (# of times/week)				0.235
Rarely 1–2 times/week ≥3 times/week	24 (45.3) 21 (39.6) 8 (15.1)		113 (57.9) 56 (28.7) 26 (13.3)	
Consumption of butter, ghee, cream (days/week)				0.002
Never/rare 1–3 days/week ≥ 4 days/week	19 (36.5) 13 (25.0) 20 (38.5)		93 (47.4) 72 (36.7) 31(15.8)	
Consumption of sweetened beverages (# of times/week)				0.331
Never 1–2 times/week ≥ 3 times/week	18 (34.0) 22 (41.5) 13 (24.5)		87 (44.8) 63 (32.5) 44 (22.7)	
Consumption of legumes (# of times/week)				0.311
Never/ rare (one or more days/week)	20 (32.7) 33 (62.3)		59 (30.4) 135 (69.6)	
Consumption of seafood (# of times/week)				0.338
Never 1–3 times/week	12 (22.6) 41 (77.4)		33 (16.9) 162 (83.1)	
Consumption of nuts (# of times/week)				< 0.001
Never 1–3 days/week	29 (54.7) 24 (45.3)		39 (19.9) 157 (80.1)	
Coffee intake (# of times/ day)				0.215
Never 1–2 times/day 3–4 times/day	16 (30.8) 25 (48.1) 11 (21.2)		43 (21.9) 92 (46.9) 61 (31.1)	
Consumption of turmeric (# of days/week)				0.015
Never 1–3 days/week 4–6 days/week	13 (25.5) 25 (49.0) 13 (25.5)		37 (18.9) 65 (33.2) 94 (48.0)	
Regular use of supplements (concentrated source of nutrients, vitamins, and minerals				0.182
No Yes	34 (64.2) 19 (35.8)		104 (53.9) 89 (46.1)	

* at least once per week over a duration of 3 months or more

** Eight or more drinks per week was regarded as heavy drinker

**Table 3:** Univariable logistic regression analysis of risk factors associated with hepatocellular carcinoma status in a hospital-based case-control study, Kuwait 2022 (both unadjusted OR and only age-adjusted OR)

Variable ^∞^	OR_unadjusted_ OR_age−adjusted_	95% CI 95% CI	*p*-value *p*-value
***Sociodemographic variables***			
Age (≥ 60 years vs. < 60 years) at enrollment in the study	4.49	2.18–9.24	< 0.001
Sex (male vs. female)	1.10 1.04	0.55–2.41 0.50–2.16	0.768 0.916
Nativity (Kuwaiti vs. non-Kuwaiti)	1.13 0.80	0.60–2.12 0.41–1.54	0.697 0.502
Employment status (unemployed vs. employed)	2.10 1.38	1.07–4.13 0.67–2.84	0.030 0.380
Education (≤ high school vs. University degree)	1.37 0.76	0.73–2.55 0.40–1.45	0.319 0.402
Total family income (KDs/ month) (≤ 1000 vs. >1000)	1.62 0.55	0.87–3.00 0.29–1.04	0.126 0.067
***Lifestyle factors and history of comorbidities***			
Family history of HCC (yes vs. no)	3.00 2.93	1.43–6.32 1.33–6.45	0.004 0.007
Parents’ blood relationship (yes vs. no)	1.17 1.29	0.61– 2.23 0.65–2.52	0.637 0.464
History of diabetes mellitus type II (yes vs. no)	1.68 1.02	0.91–0.09 0.52–1.99	0.096 0.950
History of diagnosis of HBV infection (yes vs. no)	15.01 16.55	3.01–74.69 3.03–90.53	< 0.001 0.001
History of diagnosis of HCV infection (yes vs. no)	76.97 81.83	9.87–600.24 9.87–674.82	< 0.001 < 0.001
History of diagnosis of fibrosis (yes vs. no)	588.00 708.91	141.99–243.96 136.77–3674.37	< 0.001 < 0.001
History of diagnosis of cirrhosis (yes vs. no)	1224.00 2293.14	218.03–6871.31 225.08–23362.54	< 0.001 < 0.001
History of diagnosis of non-alcohol fatty liver disease (yes vs. no)	11.42 9.91	5.69–22.90 4.86–20.23	< 0.001 < 0.001
Regular* use of nonsteroidal anti-inflammatory drugs (yes vs. no)	0.25 0.19	0.11– 0.55 0.08–0.45	0.001 < 0.001
History of diagnosis of cardiovascular disease (no vs. yes)	3.44 0.23	1.47–8.03 0.10–0.55	0.004 0.001
Regular* use of statins (yes vs. no)	0.56 0.40	0.29–1.06 0.20–0.79	0.077 0.008
Body mass index (BMI) classification			0.158
Normal body weight Overweight Obese class 1 + 2 Normal body weight Overweight Obese class 1 + 2	1.00 0.91 0.58 1.00 0.83 0.67	– 0.28–2.95 0.20–1.67 – 0.25–2.77 0.21–2.07	0.774
Daily Physical activity			0.202
Active Moderate activity Low activity Active Moderate activity Low activity	1.00 1.16 0.63 1.00 1.42 0.56	– 0.38–3.48 0.23–1.73 – 0.44–4.57 0.19–1.63	0.049
Cigarette smoking (yes vs. no)	1.34 0.78	0.69–2.62 0.39–1.57	0.382 0.493
Hookah smoking (yes vs. no)	3.72 0.37	0.85–16.25 0.08–1.65	0.081 0.191
***Dietary variables***			
Frequency of red meat consumption (days/week) (one or more days/week vs. never/ rare)	1.14 1.30	0.57–2.31 0.63–2.69	0.669 0.480
Frequency of fruits and vegetables use (days/week)			0.095
> 3 days/ week 1–3 days/week Never/ rare	1.00 2.03 2.02 1.00 3.26 5.50	– 1.00–4.14 0.21–1.40 – 0.60–17.85 1.25–24.25	
Frequency of use milk and milk substitute (glass/ week) (≤ 2 glass / week vs. ≥ 3 glass/ week)	2.99 2.64	1.33–6.00 1.15–6.05	0.006 0.021
Frequency of eggs consumption (days/week)			0.524
> 3 days/week 1–3 days/week Never/rare	1.00 0.74 1.13 1.00 0.95 1.52	– 0.34–1.57 0.54–2.34 – 0.44–2.02 0.68–3.35	0.452
Heavy alcohol drinking (yes vs. no)	5.97 6.80	1.96–18.11 2.07–22.39	0.002 0.002
Use of olive oil as a main fat source (yes vs. no)	0.35 0.30	0.17–0.70 0.14–0.61	0.003 < 0.001
Consumption of processed meat (# of times/week)			0.239
Never/rare 1–2 times/week ≥3 times/week	1.00 1.77 1.45 2.18 2.61	– 0.90–3.44 0.58–3.58 1.07–4.43 0.96–7.11	0.046
Consumption of butter, ghee, cream (days/week)			0.284
never/rare 1–3 days/week ≥4 days/week	1.00 1.45 2.06 1.00 0.96 3.70	– 0.74–2.83 0.78–5.39 – 0.43–2.12 1.67–8.22	0.002
Consumption of sweetened beverages (# of times/week)			0.335
Never/rare 1–2 times/week ≥3 times/week	1.00 1.68 1.42 1.00 2.16 2.30	– 0.83–3.40 0.64–3.17 – 1.03–4.54 0.97–5.47	0.071
Consumption of legumes (no. of times/week) (never/ rare vs. one or more days/week)	1.38 1.50	0.73–2.61 0.77–2.92	0.312 0.228
Consumption of seafood (never vs. 1–3 times/week)	1.43 1.42	0.68–3.02 0.65–3.09	0.340 0.374
Consumption of nuts (never/rare vs. 1–3 days/week)	4.86 4.69	2.55–9.26 2.39–9.20	< 0.001 < 0.001
Consumption of coffee (times/days)			0.257
3–4 times/day 1–2 times/day Never	1.00 1.50 2.06 1.00 1.67 2.22	– 0.69–3.28 0.87–4.88 – 0.74–3.73 0.91–5.43	0.211
Consumption of turmeric (days/week)			0.018
> 3 days/week 1–3 days/week Never	1.00 2.78 2.54 1.00 3.54 2.89	– 1.32–5.83 1.07–5.99 – 1.62–7.76 1.22–6.88	0.005
Regular use of supplements (yes vs. no)	0.65 0.60	0.34–1.22 0.31–1.16	0.184 0.128

* at least once per week over a duration of 3 months or more

∞ For number (%) of exposed cases and controls for each variable in final model, see Table 2

### Multivariable logistic regression model

The final multivariable logistic regression model showed the risk factors which were significantly (*p* < 0.05) and independently associated with the HCC status (Table 4). Hosmer and Lemeshow goodness-of-fit test for the final model showed a good fit (Hosmer and Lemeshow test statistic = 7.79; *p*-value = 0.454). After controlling for the effects of other variables in the model, the HCC cases compared with the controls were more likely to have had reported the history of being heavy drinker of alcohol (OR_adj_ = 14.2; 95% CI: 1.2–173.4; *p* = 0.038). Moreover, the HCC cases compared with the controls were 41.6 times more likely to have had history of NAFLD (OR_adj_ = 41.6; 95% CI: 8.9–193.5; *p* < 0.001). Furthermore, compared with the controls, the HCC cases frequently (≥ 3 glasses per week vs. rare (1–2 glasses per week or never) consumed milk or milk substitutes prior to HCC development (OR_adj_ = 7.2; 95% CI: 1.21 = − 43.4; *p* = 0.038). Conversely however, there was a significant protective effect if the participants reportedly have had regularly used NSAIDs (OR_adj_ = 0.18; 95% CI: 0.05–0.71; *p* = 0.014) or regularly consumed olive oil in their routine diet as source of fat (OR_adj_ = 0.2; 95% CI: 0.04–0.8). This study did not find any meaningful association between seafood consumption and the HCC status neither in univariable nor in multivariable logistic regression analysis.

**Table 4:** Multivariable logistic regression model of risk factors associated with the hepatocellular carcinoma status in a hospital-based case-control study, Kuwait 2022

Variable^∞^	Adjusted odds ratio**	95% confidence interval	*p*-value
Heavy alcohol drinking (yes vs. no)	14.2	1.2–173.4	0.038
Frequency of use of milk and milk substitute (≥ 3 glass/ week vs. never/ rare (1–2 glass)	7.2	1.2–43.4	0.030
History of NAFLD*** diagnosis (yes vs. no)	41.6	8.9–193.5	< 0.001
Regular* use of olive oil in diet as fat source (yes vs. no)	0.17	0.04–0.8	0.020
Regular use of NSAIDs*** *(yes vs. no)	0.20	0.05–0.71	0.014

Hosmer and Lemeshow test statistic = 7.79, *p* = 0.454

* at least once per week over a duration of 3 months or more

**Estimates are adjusted for effects of infection with hepatitis B virus and hepatitis C virus, age (completed years) at enrollment employment status, total family income (Kuwaiti Dinars per month), consumption of butter, ghee, cream (number of days/ week), consumption of turmeric (number of days/ week)

*** NAFLD = non-alcoholic fatty liver disease

## Discussion

This study examined the relationship between sociodemographics, dietary patterns, tobacco smoking, alcohol drinking, family history of HCC, comorbidities including diagnosis of infection with HBV, HCV, diabetes type 2, NAFLD, use of NSAIDs, statins and HCC risk. The final multivariable logistic regression model revealed that the history of NAFLD diagnosis, heavy alcohol drinking, frequency of use of milk and milk substitutes, regular use of NSAIDs and regular use of olive oil as a source of fat in the diet were significantly and independently associated with HCC risk after the adjustment for the effects of other variables in the model.

Alcohol consumption was associated with an increased HCC risk in this case-control study. Previously, a meta-analysis of 11 case-controls studies and another meta-analysis of 16 cohort studies revealed that heavy alcohol drinkers had a significantly higher HCC risk compared with nondrinkers or light drinkers of alcohol [29]. The plausible concurrent and/ or alternate pathobiological mechanisms that could potentially explain the association between heavy alcohol drinking and HCC risk include (i) metabolic product of alcohol is acetaldehyde, that has the potential of inducing oxidative stress, thus theoretically generating DNA adducts (i.e., DNA segments) bound to cancer causing chemical. This process could lead to the development of cancerous cells, or carcinogenesis [30]; (ii) heavy alcohol drinking modifies enzymes alcohol dehydrogenase1B (ADH1B) and aldehyde dehydrogenase 2 (ALDH2), hence promote liver carcinogenesis [31]; (iii) heavy alcohol consumption and long-term exposure to alcohol may lead to alcoholic liver disease, which lead to the liver cirrhosis and eventually progress to HCC [32]; (iv) alcohol as a solvent could enhance penetration of carcinogens inside the cells [33]; and/ or heavy alcohol consumption could impair immunity and hepatic detoxification [32].

The results of this case-control study showed that NAFLD (NASH - nonalcoholic steatohepatitis) was significantly and independently related to an increased HCC risk. A review and meta-analysis of 103 observational studies showed a significant association between NAFLD/NASH and HCC risk [34]. Clinically, NAFLD and NASH are caused by the multiple risk factors [35]. Adipose tissue secrete adipokines that provide fatty acid – a main source of NAFLD development. Several adipokines including resistin, leptin, and visfatin are known to play a role in the development of NAFLD, progression to NASH and subsequently to HCC [36, 37]. However, ghrelin, adiponectin, and irisin are considered to have beneficial impact on NAFLD and NASH [38, 39].

In this case-control study, milk and milk substitutes were positively associated with the increased HCC risk. A systematic review and dose-response meta-analyses of seven cohort and eight case-control studies showed that milk and milk substitutes were positively associated with liver cancer [40, 41]. Moreover, two cohort studies showed that consumption of milk and milk substitutes (but not the yogurt) were significantly associated with increased HCC risk [42, 43]. Numerous mechanistic pathways have been proposed to concurrently and/or alternatively explain the association between excessive consumption of dairy products and increased HCC risk. *First*, several observational and experimental studies have found milk and milk substitutes increase the concentration of insulin-like growth factor-1 (IGF-1), and high levels of IGF-1 could enhance the cancer risk by inhibiting apoptosis and stimulating cell proliferation [44]. This effect was confirmed by meta-analysis of eight randomized trials and 15 cross-sectional studies [45]. In experimental studies, high levels of IGF-1 were shown to enhancing the development of certain cancers including HCC [46, 47]. Furthermore, liver is the main source of circulating IGF-1, therefore compared to other organs, liver has increased exposure to this hormone. High levels of IGF-1 may enhance the growth of HCC both in vivo and vitro [48]. *Second*, there is an evidence that excessive amount of branched-chain amino acids, lactose which is disaccharide of milk and milk substitutes provide galactose with IGF-1, which could possibly enhance and stimulate the mechanism of target rapamycin complex 1 signaling, and possibly leading to enhance cell proliferation and carcinogenesis [49]. Additionally, milk and milk substitutes are essential sources of dietary fat and excessive consumption of fat has been shown to be correlated with insulin resistance which could be linked to liver diseases including elevated HCC risk [50, 51].

In this case-control study, regular use of olive oil had a significant and independent protective against the HCC risk. A systematic review and meta-analysis showed that the consumption of regular olive oil in the diet as a source of fat was associated with 31% lower cancer risk [52]. Moreover, a large cohort study and a case-control study showed that the regular intake of monosaturated fatty acid was associated with lower HCC risk [53, 54]. A systematic review and a meta-analysis of 117 studies (a total 3,202,496 participants) showed a significant inverse association between Mediterranean diet and the HCC risk [55–57]. Mediterranean diet is considered a strong method to fight cancer and this protective effect has been extensively reported [58]. This diet is based on high intake of monounsaturated fatty acid from extra virgin olive oil, fruits and vegetables, nuts, fish, legumes, reducing the consumption of dairy products and meat [56]. The monounsaturated fatty acid down regulates nuclear factor i.e., kappa B NF-κB, which is a family of proteins that facilitate cell functions [59]. NF-κB may be found in an excessive amount in some types of pre-cancerous cells, eventually leading to cancer cell growth [60].

Regular use of NSAIDs had a significantly protective effect against HCC risk in this study. A systematic review and meta-analysis of the observational studies showed that aspirin use was significantly associated with lower HCC risk [61]. Furthermore, regular NSAIDs use was shown to be associated with 41–54% reduction in HCC risk [62, 63]. The use of NSAIDs tends to lower the incidence and related mortality of certain malignancies, especially gastrointestinal cancer. Chronic inflammation is believed to induce the pathogenesis of HCC [64]. Cyclooxygenase (COX) enzymes are well-known targets of NSAIDs. However, the conventional NSAIDs non-selectively inhibit both the constitutive form COX-1, and the inducible form COX-2. COX-2 facilitates inflammatory processes and its expression is undetectable in most normal tissues. It is usually overexpressed in response to proinflammatory stimuli, such as proinflammatory cytokines, mitogens, tumor promoters and growth factors including those which facilitate hepatic carcinogenesis [64–66]. NSAIDs tend to obstruct and alter the COX enzymatic pathways important in prostaglandins synthesis which lead to inhibition of HCC cell growth by cell cycle induction of apoptosis. NSAIDs use may regulate hepatocarcinogenesis by non-COX pathways like mitogen-activated protein kinase and PI3K/Akt pathways [67], Moreover, NSAIDs use can downregulate the pro-inflammatory cytokines [65].

This case-control study did not show a meaningful relationship between the consumption of seafood and the HCC risk, whereas, EPIC cohort study [68], and a nested case-control study showed that regular consumption of fish was inversely associated with the HCC risk [69]. The non-significant association between fish consumption and HCC risk in this study could possibly be due to a smaller sample size. Future studies may consider this observation at the planning stage.

### Strengths and limitations of the study

#### Strengths

The study has some notable strengths including, (i) HCC cases enrolled were a representative sample of all such cases in Kuwait. In this study, we enrolled all alive HCC cases using the list frame of the patients from (2018–2022) KCCC; (ii) all the studied HCC cases were diagnosed in a relatively narrow time window, therefore, presumably were comparable with the incident HCC cases and this attribute of selected HCC cases might have contributed to minimizing the recall bias in exposures assessment.

#### Limitations

This study has some limitations; *First*, HCC is a rare and aggressive disease with a median survival time of approximately 6–20 months, thus, such incident HCC cases could not be enrolled. Therefore, the possibility of prevalence-incidence (Neyman’s) bias could not be ruled out. Furthermore, we did not have the data on the specific date of HCC diagnosis, consequently we could not calculate and report the median survival time of cases till their enrollment in the study. *Second*, the data were collected in-person interviews both from cases and controls. Thus, the possibility of respondent’s bias may not be ruled out, since the respondents might have withheld answers to some of the questions pertaining to lifestyle factors including alcohol consumption, body weight, tobacco use. However, the interviewers were trained in interviewing technique and were careful about this aspect of exposures’ assessment. Hence, interviewers tried their best to pose the questions to cases and controls in a comparable manner to ensure unbiased exposures’ assessment. *Third*, this was a hospital-based case-control study, therefore, the role of Berkson’s bias cannot be ruled out. This bias tends to make risk factors’ distributions similar between case and control groups, thus, weakening the associations of interest between the risk factors of interest and outcome (case-control status). However, we tried to ensure the enrollment of controls free from any gastrointestinal ailments. *Fourth*, all the HCC cases in this study were histopathologically confirmed, while the study controls didn’t undergo the similar rigorous diagnostic/ screening procedures to rule out the HCC positive status, rather their HCC status was self-reported. Such disparity in diagnostic/ screening procedures for disease status assessment in cases and controls might have led to information bias in the data. However, we selected the controls from among the individuals who were visiting the general hospitals for seeking healthcare for medical conditions other than that of gastrointestinal tract ailments. Therefore, the possibility of such information bias in the data is likely to be very minimal. *Fifth*, the controls were selected from the patients with minor ailments other than those of gastrointestinal tract who were visiting outpatient clinics of the tertiary-care hospitals. Thus, the controls might not have truly represented the exposures’ experience of the general population at large, hence potentially hampering the generalizability of the results to the target population. *Sixth*, some confidence intervals for the effect estimates are relatively imprecise mainly because of limited availability of cases due to the aggressive nature of the disease. Moreover, owing to a relatively small sample size and the resultant low study power, some of suspected exposure-outcome associations might have remained undetected. Future studies may take into account this aspect at a planning stage. *Lastly*, some of the cases soon after HCC diagnosis travelled abroad for treatment, therefore, we might have missed out such cases from the enrollment in this study. However, the proportion of such HCC patients presumably was not large enough to impair the parameters’ estimates in this study.

## Conclusions

In conclusion, heavy alcohol consumption, non-alcoholic fatty liver disease, frequent consumption of milk and milk substitutes were associated with a significantly increased HCC risk. Conversely however, the regular use of olive oil in the diet as a source of fat, and the regular use of NSAIDs were significantly protective against the HCC risk. Adapting healthy dietary habits and preventing/ treating NAFLD may minimize the HCC risk. Future research with a larger sample size may contemplate validating the results of this study and unraveling additional risk factors contributing to the HCC risk. The resultant data may help design and implement evidence-based educational programs for the prevention of HCC in this and other similar settings.

## Data Availability

The dataset used in this study can be made available on a reasonable request to the corresponding author (SA).
